# Brain–Heart Interaction and the Experience of Flow While Playing a Video Game

**DOI:** 10.3389/fnhum.2022.819834

**Published:** 2022-04-28

**Authors:** Shiva Khoshnoud, Federico Alvarez Igarzábal, Marc Wittmann

**Affiliations:** ^1^Institute for Frontier Areas of Psychology and Mental Health, Freiburg, Germany; ^2^Department of Neurosurgery and Neurotechnology, Institute for Neuromodulation and Neurotechnology, University of Tübingen, Tübingen, Germany

**Keywords:** experience of flow, heart-evoked potential, brain–heart interaction, self-referential processing, arousal, time perception

## Abstract

The flow state – an experience of complete absorption in an activity – is linked with less self-referential processing and increased arousal. We used the heart-evoked potential (HEP), an index representing brain–heart interaction, as well as indices of peripheral physiology to assess the state of flow in individuals playing a video game. 22 gamers and 21 non-gamers played the video game Thumper for 25 min while their brain and cardiorespiratory signals were simultaneously recorded. The more participants were absorbed in the game, the less they thought about time and the faster time passed subjectively. On the cortical level, the fronto-central HEP amplitude was significantly lower while playing the game compared to resting states before and after the game, reflecting less self-referential processing while playing. This HEP effect corresponded with lower activity during gameplay in brain regions contributing to interoceptive processing. The HEP amplitude predicted the level of absorption in the game. While the HEP amplitude was overall lower during the gaming session than during the resting states, within the gaming session the amplitude of HEP was positively associated with absorption. Since higher absorption was related to higher performance in the game, the higher HEP in more absorbed individuals reflects more efficient brain–heart interaction, which is necessary for efficient game play. On the physiological level, a higher level of flow was associated with increased overall sympathetic activity and less inhibited parasympathetic activity toward the end of the game. These results are building blocks for future neurophysiological assessments of flow.

## Introduction

**FIGURE 1:**
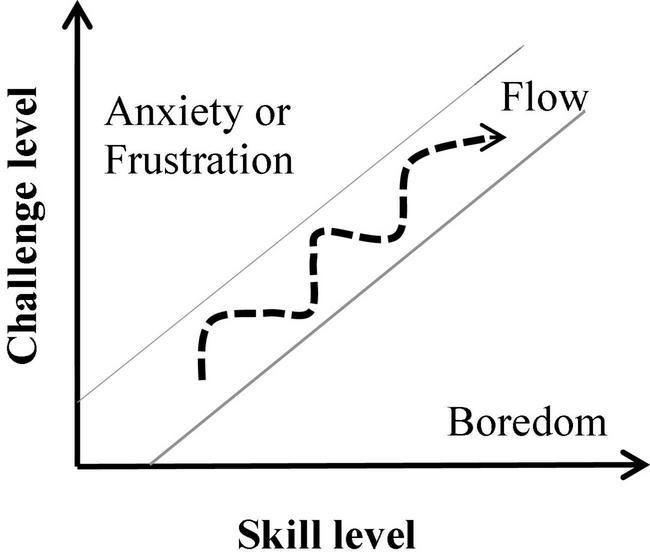
The flow-channel model (Csikszentmihalyi, 1975).

Csikszentmihalyi (1975) described the state of flow as an optimal experience that occurs when an individual is immersed in an activity and feels enjoyment. The main antecedents of this experiential state are clear goals, immediate feedback, and an optimal balance between the individual’s skills and the level of challenge posed by the activity (the skill-challenge balance; Csikszentmihalyi and Csikszentmihalyi, 1992; Engeser and Rheinberg, 2008; Keller and Blomann, 2008; Fong et al., 2015). According to the flow-channel model (Csikszentmihalyi, 1975, 1990; Figure 1), whenever the challenge level of the activity outweighs the performer’s skill level, the person will become frustrated or anxious. In contrast, when the level of challenge is lower than the individual’s skill level, they will feel bored. The flow experience has been associated with successful performance and feelings of competence (Engeser and Rheinberg, 2008; Jin, 2012; Rutrecht et al., 2021), since frustration and boredom lead to diminished concentration and, consequently, poor performance (Perone et al., 2019). Flow is often linked with the experience of losing the sense of time and of the self (Csikszentmihalyi and Csikszentmihalyi, 1992; Wittmann, 2015, 2018; Rutrecht et al., 2021).

The underlying physiological and neural mechanisms of the flow state are beginning to be studied extensively. Researchers have attempted to map specific neurophysiological indicators of this mental state by employing a skill-challenge balance. So far, the results have been heterogeneous and inconclusive in terms of identifying a unified mechanism underlying this state (for more details see our review, Khoshnoud et al., 2020). In the present study, we focused on two important mechanisms of the experience of flow, namely heightened arousal as manifested in elevated cardiovascular (de Manzano et al., 2010; Chanel et al., 2011; Keller et al., 2011; Tian et al., 2017; De Sampaio Barros et al., 2018) and loss of self-related processing typically associated with decreased activity in the default-mode network (DMN, Ulrich et al., 2014, 2016, 2018; De Sampaio Barros et al., 2018).

Video games are promising tools for systematically inducing flow under controlled laboratory conditions. They offer clear goals, immediate feedback, and challenging tasks with the possibility of modulating the level of difficulty for achieving the desired skill-challenge balance (Salen and Zimmerman, 2003; Alvarez Igarzábal, 2019). For this reason, we chose to investigate arousal and self-related processing during flow states with the commercially available video game *Thumper*, which our previous research had shown to be highly flow inducing (Rutrecht et al., 2021). We tested experienced gamers and non-gamers who played the game for 25 min and recorded their electroencephalography (EEG) and cardiorespiratory signals. Gamers were recruited because their experience with video games would in principle allow them to more easily familiarize themselves with the game and enter a flow state. The non-gamer sample constituted the control group to allow us to compare how individuals lacking gaming skills would experience the play sessions – i.e., if they would enter a state of flow or not. This study was conducted in the context of the VIRTUALTIMES project with the goal of developing a therapeutic game that could reduce the symptoms of patients with psychiatric disorders through the induction of flow states. Recent conceptualizations suggest that inducing the experience of flow with video games could help reduce pathologically increased self-rumination (Kühn et al., 2018), which is associated with time distortions in many patient groups with psychopathologies, such as depression, attention deficit hyperactivity disorder, and addiction (Wittmann et al., 2007; Khoshnoud et al., 2018; Vogel et al., 2018). The game resulting from the VIRTUALTIMES project should effectively induce flow states regardless of the gaming experience of the player. We included the non-gamer sample for this reason as well, since potential differences between groups (or a lack thereof) would inform the design of the game. Continuous play, which is the more natural way of playing video games – by progressing and encountering increasingly difficult challenges – was preferred over difficulty modulation, in which the level of challenge is adapted to the player’s performance. Employing such form of difficulty modulation to achieve the skill-challenge balance can facilitate flow induction, but does not necessarily lead to a state of flow.

### Flow, Selflessness, and Brain–Heart Interaction

Reduced self-awareness is one of the main characteristics of the flow experience (Csikszentmihalyi, 1975, 1990). The high levels of concentration and focused attention demanded by the task at hand restrict resource allocation for task-irrelevant demands, like body- and self-awareness. Several studies reported less self-referential processing during the flow experience by showing deactivation of the DMN, specifically the medial prefrontal cortex (MPFC) (Sadlo, 2016; Ulrich et al., 2016, 2014, 2018; De Sampaio Barros et al., 2018; Ju and Wallraven, 2019). DMN activity is associated with relaxation, mind-wandering, and self-referential thinking, and it diminishes during task-focused and goal-directed actions (Raichle et al., 2001; Goldberg et al., 2006). Increased activation of the insular cortex has also been reported during flow states (Ulrich et al., 2016; Huskey et al., 2018; Ju and Wallraven, 2019). The insular cortex is the primary visceral area (Craig, 2009). Recent neuroscientific research has shown that cortical processing of signals from internal visceral organs, like the heart and the gut, are important for cognition (Park et al., 2014; Critchley and Garfinkel, 2018), self-consciousness (Damasio and Carvalho, 2013; Tallon-Baudry et al., 2018; Park and Blanke, 2019), and subjective time (Craig, 2009; Wittmann, 2013; Teghil et al., 2020). According to Tallon-Baudry et al. (2018), the gut and the heart are similar to ticking clocks that constantly send intrinsically generated information up to the central nervous system. By monitoring this bodily information, the brain creates a neural reference frame for developing a first-person perspective.

To evaluate self-related processing during moments of flow, we investigated the brain–heart interaction by exploring possible associations between the heart-evoked potential (HEP) – an index representing the neural processing of cardiac afferents – and the flow experience while playing the game. HEPs are cortical electrophysiological responses in the brain that are time-locked to the R-peaks of the simultaneously measured electrocardiography (ECG) signal (Montoya et al., 1993; Schandry and Montoya, 1996). The precise pathways underlying the HEP are unknown. In addition to cardiac and blood vessel receptors, it has been argued that cardiac afferents are projected to the cortex through tactile and proprioceptive receptors (Azzalini et al., 2019; Park and Blanke, 2019). Research has shown that self-relatedness (Babo-Rebelo et al., 2016, 2019; Park et al., 2016; Sel et al., 2017), focus of attention (Petzschner et al., 2019), emotional feelings (Fukushima et al., 2011; Shao et al., 2011), and arousal (Luft and Bhattacharya, 2015) modulate the HEP amplitude.

Despite the established relationship between selfhood and the HEP amplitude, the findings regarding the direction of this association are mixed; i.e., there are positive and negative correlations between the HEP amplitude and self-relatedness. Employing a full-body illusion paradigm, Park et al. (2016) identified a relationship between the HEP amplitude over fronto-central scalp sensors and experimentally modulated changes in self-identification. The negative HEP amplitude was more pronounced during the condition with lower self-identification rating compared to the condition with higher self-identification. A study conducted by Babo-Rebelo et al. (2016) revealed a direct link between self-relatedness of spontaneous thoughts and the HEP amplitude. The HEP amplitude was evaluated in two conditions: one where participants were the subject (first-person perspective) of presented thoughts (“I” as in “I like him”) and another where they were the object of the thoughts (“Me” as in “He likes me”). The amplitude of the HEP in the mid-posterior regions of the brain co-varied negatively with the engagement of “I”, showing higher amplitudes for lower ratings on the “I” scale. The HEP amplitude also differed between “high” and “low” trials on the “Me” scale over medial frontal sensors with higher HEP amplitudes for the trials with higher score of the “Me” scale.

In the present work, we investigated the link between flow and the HEP amplitude as an index reflecting self-related processing. This was the first time that the HEP was studied in the context of flow, as far as we know. Considering the connection between the HEP amplitude and the self (Park et al., 2016; Babo-Rebelo et al., 2019, 2016), as well as the existing overlap between the cortical sources of the HEP and the DMN (Park and Blanke, 2019), one can expect to see a decrease in neural responses to the heartbeats and a lower HEP amplitude while playing a video game compared to the resting state. This means that self-referential processing is lower while gaming than while resting (*Hypothesis1*). The aforementioned associations between selfhood and the amplitude of the HEP may refer to a distinct HEP during gameplay as a result of experiencing flow and a loss of self-awareness. Considering the mixed nature of the direction of the reported associations, we expected a relationship between flow and the HEP amplitude without making any assumptions regarding its direction (*Hypothesis 2*).

### Flow and Arousal

The feeling of enjoyment along with high levels of concentration on a given task suggests that flow states are modulated through arousal levels, which are related to the activation of the autonomic nervous system (ANS). The two branches of the ANS, the sympathetic nervous system (SNS, responsible for “fight-or-flight” responses) and the parasympathetic nervous system (PNS, responsible for “rest-and-digest” responses) operate as excitatory and inhibitory physiological mechanisms, respectively (Cacioppo et al., 2007). Numerous indicators of sympathetic and parasympathetic activity have been studied in flow research, including cardiovascular, electrodermal, and respiratory measures. Aggregating the existing empirical results, both linear and non-linear associations between the activity of the autonomic nervous system and flow have been reported (Khoshnoud et al., 2020). The most established theory links the experience of flow to high levels of the SNS activity (Kivikangas, 2006; de Manzano et al., 2010). The reported positive association between the electrodermal activity (EDA) as a robust indicator of sympathetic arousal (Critchley and Nagai, 2013) and flow (Nacke and Lindley, 2010; Léger et al., 2014; Ulrich et al., 2016, 2014) confirms this theory. Increased flow while playing a video game was reported to be related to an increased heart rate (de Manzano et al., 2010; Bian et al., 2016), a faster respiration rate (Bian et al., 2016; Tian et al., 2017), high levels of salivary cortisol (Keller et al., 2011), and lower heart-rate variability (HRV) measures, specifically lower high-frequency (HF) HRV (Chanel et al., 2011; Keller et al., 2011; Harmat et al., 2015; Harris et al., 2016; De Sampaio Barros et al., 2018; Kozhevnikov et al., 2018). HF-HRV reflects the variance in the 0.15–0.4 Hz frequency range of the heart rate and is a reliable indicator of parasympathetic activity (Laborde et al., 2017; Shaffer and Ginsberg, 2017).

In two studies, the reported heightened sympathetic activity during flow was associated with the modulated parasympathetic activity manifested by deeper respiration (de Manzano et al., 2010; Harmat et al., 2015; Tian et al., 2017). According to Porges’ (1995, 2021) polyvagal theory, parasympathetic influences are essential for an individual’s successful adaptation to changing environmental demands. Several studies have suggested a connection between parasympathetic activity and cognitive performance, working memory, and attention (Hansen et al., 2003; Frewen et al., 2013; Mahinrad et al., 2016; Tsakiris and De Preester, 2018). The so-called parasympathetic modulation of sympathetic activity may describe flow as a state of heightened arousal accompanied by a feeling of pleasure and, at the same time, seemingly paradoxical relaxation, which is suggested to act as a physiological coping mechanism for high demands of attention in moments of flow (de Manzano et al., 2010; Ullén et al., 2010).

In light of the above-mentioned findings, we expected to see a positive association between sympathetic activity and the self-reported flow measures. Given the reported connection between parasympathetic activity and attention, relatively increased parasympathetic activity should help cope with the changing attentional demands of the task. We assumed that more flow would be associated with increased sympathetic and parasympathetic activity (*Hypothesis 3*).

## Materials and Methods

### Participants

Participants were 43 healthy subjects (Gamers: *n* = 22; 19 males, 3 females; Non-Gamers: *n* = 21; 18 males, 3 females) with an average age of 24.90 ± 3.98 years (Gamers: 25.59 ± 4.56, Non-Gamers: 24.19 ± 3.23). The age difference between the two groups was not significant (*t*(41) = –1.15, *p* = 0.254). All participants were recruited via online platforms (Student Services University Freiburg), flyers, word-of-mouth dissemination, and through advertisements on social media and gaming forums. As an inclusion criterion for being a gamer, individuals had to: (a) consider themselves a gamer, (b) have more than five years of experience playing videogames regularly, and (c) have played videogames in the past six months for at least 5 h a week. On average, gamers had 15.36 ± 4.97 years of experience and non-gamers, 6.28 ± 7.3 years with an average of 15.97 ± 8.89 and 0.48 ± 0.54 h of playing games in a week, respectively. Gaming experience in years of playing (*t*(41) = –4.77, *p* < 0.001) and average hours of gameplay in a week (*t*(40) = –7.96, *p* < 0.001) were significantly higher in gamers than in non-gamers. No significant difference was identified for the educational level between the two groups (*U* = 263, *p* = 0.4). This study was approved by the local Ethics Committee of the Institute for Frontier Areas of Psychology and Mental Health (IGPP_2019_01).

### Questionnaires

The state of flow was measured with the 10-item *Flow Short Scale* (FSS, Rheinberg and Vollmeyer, 2003) with seven-point Likert scales ranging from 1 (not at all) to 7 (very much) which are linked to two subscales: absorption and fluency. *Absorption* is measured by four items (e.g., “I am totally absorbed in what I am doing” or “I don’t notice time passing”), while the remaining six items index the *fluency* of performance (e.g., “My thoughts/activities run fluidly and smoothly” or “The right thoughts/movements occur of their own accord”). Considering the flow-channel model (Figure 1), the absorption subscale can be considered a stronger indicator of flow, while fluency can also occur in situations in which external demands are lower than individual skills (Peifer et al., 2014). Besides the overall flow score comprising all items (FSS mean score), we also looked at the absorption and fluency subscales’ mean values separately in their relationships with the other behavioral and physiological measures. The Cronbach’s alpha values found in our dataset for the FSS mean score, the absorption, and the fluency subscales are 0.847, 0.768, and 0.787, respectively.

We used the *Subjective Time, Self, and Space* (STSS) questionnaire (Jokic et al., 2018) to assess the effects of the gaming session on time and bodily perception. This questionnaire consists of an item to evaluate bodily awareness (“How intensively did you experience your body most of the time?”) with a non-verbal pictorial scale with the answer category ranging from 1 to 7. Three items assess the experience of time: (1) “Intuitively, without thinking about it, the gameplay session lasted ____ minutes and ____ seconds” (estimated duration); (2) “How often did you think about time?” (thinking about time); and (3) “How fast did time pass?” (speed of time passage). The latter two time questions were answered with a vertical streak on a visual analog scale (a 10-centimeter-long horizontal line) ranging from “not at all” to “a lot” on question two and from “very slowly” to “very fast” on question three. The *Self-Assessment Manikin* (SAM) scale (Bradley and Lang, 1994) was used to measure *valence* (SAM-valence) and *arousa****l*** (SAM-arousal) changes with non-verbal, pictorial scales after vs. before the gaming session. We used the 5-point version of the scales.

### Experimental Design

The study took place on 3 consecutive days. The first two days were training sessions for participants to learn how to play the chosen game and on the third day the participants’ EEG and cardiorespiratory signals were recorded simultaneously while they played the game. The video game we used was *Thumper*, an action/rhythm game developed by the studio Drool and released in 2016. We acquired and launched the game through the platform Steam. In this game, the player controls a silver beetle that moves forward automatically on a track within an abstract landscape. The player watches from behind the beetle, so that s/he can react to different elements that are placed on the track. The track also presents the player with obstacles like sharp curves or spikes that can damage the beetle if they are not averted in time. Players can listen to the rhythm induced by the soundtrack to help with the timing of these actions. The goal of this game is to achieve the highest score possible in each level and level section by hitting as many lights as possible and avoiding collisions with obstacles.

The first session lasted 90 min in which participants were asked to read the information sheet, sign the consent form, and answer questions regarding their well-being. After that the experimenter explained the gameplay basics to the participants and let them play for about 60 min, starting from the first level of the game. In the second training session, participants played the game for another 60 min, starting from the level they had reached in the previous session, unless they preferred to start at a lower level. In the final recording session, participants could choose the level at which they felt comfortable to start. We recommended that they neither pick a level that was too challenging nor one that was too easy for them. This was done to increase the chances that they would enter a flow state during the gameplay session. Gamers and non-gamers therefore played at different game levels according to their skills, since higher levels are more challenging than lower ones.

In the final session, participants were asked to sit in a comfortable chair watching a screen positioned 70 cm in front of them in an electrically shielded room. They were requested to fill out the SAM questionnaire after all the electrodes had been placed. The recording session had three stages: a pre-game resting-state period of 5 min with eyes open, followed by 25 min of gameplay, and then another 5 min with a post-game, resting-state recording. Participants were asked to play *Thumper* continuously for 25 min, since there seems to be an adaptation period while playing after which participants tend to lose track of time (Tobin et al., 2010; Bisson et al., 2012). Yun et al. (2017) reported that 87.5% of the participants in their study required at least 25 min to get into the flow state. After the post-game session, participants filled out the SAM questionnaire once again, as well as the post-task questionnaires STSS, and FSS. After the task, they watched their own recorded gameplay and answered questions regarding their subjective experience.

### Signal Recordings

Continuous EEG signals were recorded using a 32-channel electrocap (ActiCHamp, BrainVision) with active electrodes positioned according to the extended 10–10 international system. All electrodes were referenced to the Fz electrode, with the ground electrode placed on the forehead. Electrode impedances were kept below 10 kΩ. EEG signals were digitized with a 1000 Hz sampling rate and band-pass filtered within the 0.01–120 Hz range. One electrocardiogram (ECG) signal was acquired using three Ag/AgCl electrodes, which were positioned according to the Lead II Einthoven configuration: two electrodes placed on the right clavicle and the left hip/abdomen (active electrodes), and one electrode placed on the left clavicle (ground electrode). A respiratory signal was acquired using the Brain Vision respiration belt attached to the participant’s chest or abdomen, depending on the subject’s breathing mode. All peripheral signals were co-registered with the EEG via the auxiliary inputs of the amplifier.

### Data Processing

#### Electroencephalography Analysis

The recorded EEG signals were processed with the Matlab software using custom-written scripts and the EEGLAB toolbox functions. The raw EEG signals were first down-sampled to 250 Hz and filtered with a band pass filter of 1.5–70 Hz. After this initial filtering step, line noise and other large non-stationary artifacts were identified and cleaned using the artifact subspace reconstruction (ASR) approach (Chang et al., 2020). The cleaned signals were re-referenced to a common average reference. To identify other non-brain related EEG contamination (e.g., eye-blinks, muscle, heart, and channel noise), we used the adaptive mixture independent component analysis (AMICA; Palmer et al., 2006, 2011, 2008) to decompose the EEG signal into its independent components (ICs). AMICA has shown superior performance among the blind-source separation algorithms for EEG decomposition (Delorme et al., 2012). After decomposing the signals into ICs, their equivalent current dipoles were also computed using the three-shell, boundary-element-method head model based on the MNI brain template using the DIPFIT plugin of the EEGLAB toolbox^1^. The identified ICs were then automatically classified and labeled using a machine-learning approach, which has been trained to classify the ICs based on several characteristics, such as spectral properties and brain topography (Pion-Tonachini et al., 2019). The brain-related ICs, which were labeled with the probability higher than 0.5 and had a dipole residual variance less than 0.2, were selected for further analysis. This procedure cleans the signal from eye-blinks, muscle noise, heart artifact, and other contamination and preserves pure brain-related activity. To calculate the average alpha frequency band (8 – 12 Hz) power, we used the Welch’s power-spectral density estimation method (using the hamming window with the size of 2000 samples and 10% overlap between windows).

#### Electrocardiography Analysis

The raw ECG signals were imported to the Kubios Heart Rate Analysis Software (Kubios, Inc., University of Western Finland, Finland) to calculate inter-beat intervals (IBIs) of successive heartbeats and associated heart-rate variability measures. Recordings were first screened manually for ectopic and missing beats, after which the appropriate artifact-removal threshold available in Kubios was applied. The average IBI was calculated for each condition and each subject. Power-spectrum analysis was performed using the Fast Fourier Transformation (FFT) method provided by the software, and then the log power of the low-frequency HRV (LF-HRV; 0.04–0.15 Hz) and the high-frequency HRV (HF-HRV; 0.15–0.4 Hz) were computed. Since the HF-HRV is influenced by breathing rates below 9 or above 24 cycles per minute (Malik et al., 1996; Laborde et al., 2017), we used the obtained respiration rate from the respiratory signal to control for respiratory rates in these ranges. Participants whose breathing rates exceeded these limits were excluded from the frequency-domain HRV analysis.

#### Respiration Analysis

The recorded respiratory signals were first down sampled to 25 Hz and lowpass filtered using an infinite-impulse-response (IIR) lowpass filter with the order of 8 and passband frequency of 2 Hz. Since the respiratory signals were contaminated with large artifacts due to movements, the signal amplitudes outside the chosen threshold (mean ± 2 × S.D.) were replaced with the interpolated values. Then the local maxima were identified and the average respiration rate (RR-mean) and the standard deviation of the respiration rate (RR-STD) were calculated.

#### Heartbeat-Evoked Potentials

For the HEP analysis, cardiac R peaks of the ECG signal were detected offline and used as triggers for the EEG segmentation of the pre-processed EEG signals. The R-peak detection was performed by decomposing the ECG signals using discrete wavelet transform analysis with the “sym4” wavelets. The “sym4” wavelet resembles the QRS complex of ECG signals, which makes it a good choice for the QRS detection. Wavelet decomposition was performed for five levels, and then the signal approximation was built from the wavelet coefficients of levels 4 and 5. The exact location of R peaks was identified using the squared absolute values of the signal approximation and a peak-finding algorithm. EEG epochs were then extracted time-locked from 200 ms before to 600 ms after the detected R-peaks. Baseline correction was performed from –200 to –100 ms time-locked to the R peak for each epoch. The HEP signals were then averaged for each electrode, each condition, and each subject.

#### Source Localization

Cortical source reconstruction and surface visualization were implemented by the BrainStorm toolbox (version January 04, 2021; Tadel et al., 2011) on the Matlab software. After co-registration of the EEG sensors and the template anatomy (MNI brain template, ICBM152) for each participant, the forward model was calculated using the boundary element method from the open-source software OpenMEEG (Gramfort et al., 2010) on the cortical surface of the template brain. Cortical currents for each subject and each condition were estimated by a distributed model consisting of 15,002 current dipoles using the weighted minimum-norm, current-estimation method with the dynamical statistical parametric mapping (dSPM) providing z-score cortical currents. The orientation of dipoles was considered constraint and normal to the cortex. The cortical currents were then spatially smoothed (7 mm) and averaged over 400 to 500 ms after the R-peak (in which a significant difference between the pre-game and the game was found). The anatomical description is based on the Desikan–Killiany (Desikan et al., 2006) and the Brodmann parcellations available in the BrainStorm toolbox.

### Statistical Analysis

(1) For between-subject behavioral comparisons, we computed the independent *t-*test and, in non-normal distributions, the Mann-Whitney *U*-test. (2) Peripheral measure comparisons were performed using repeated-measure ANOVAs with condition (pre-game, game, and post-game) as a within-subject factor, group (gamers vs. non-gamers) as a between-subject factor, and each measure as a dependent variable. Degrees of freedom in repeated-measure ANOVAs were corrected according to the Greenhouse-Geisser method for violation of the sphericity assumption when appropriate. (3) Correlations were analyzed with Pearson’s correlation coefficients (*r*) affording parametric assumptions, such as a normal distribution. Whenever we found a significant effect using the Shapiro-Wilk test for non-normality in one of the two variables, we reported the Spearman correlation coefficient (ρ). Analysis significance levels were set to *p* < 0.05 for each correlation. The false-discovery rate (FDR) method, a multiple-comparisons-correction procedure by Benjamini and Hochberg (1995) was used to control for multiple tests for each of the correlation tables.

(4) Differences in the HEPs between conditions and the corresponding cortical sources were tested using two statistical methods. One was the cluster-based permutation *t*-test implemented in the Fieldtrip toolbox, which is available in the BrainStorm toolbox. With this procedure, individual samples with a *t* value higher than threshold (*p* < 0.05, two tailed), are clustered in connected sets based on temporal and spatial adjacency. For each cluster, cluster-level statistics are assigned by taking the sum of the *t* values within a cluster, and then the maximum of the cluster-level statistics is selected for the evaluation of the null hypothesis. After shuffling the condition labels 10,000 times, the two-tailed Monte-Carlo *p* value corresponds to the proportion of elements in the distribution of shuffled, maximal cluster-level statistics that exceeds the observed maximum or minimum original cluster-level test statistics. As this method uses maximal cluster-level test statistics, it intrinsically controls for multiple comparisons in time and space (Park et al., 2014). Without *a priori* selection of a time window and region of interest, getting a significant result from this method does not establish significance of effect for latency or location (Sassenhagen and Draschkow, 2019). To test the differences of the HEP amplitude in the sensory domain for which we did not have an *a priori* assumption of a time window and scalp locations, we used a non-parametric permutation test with an FDR correction for multiple comparisons. After finding the time window in which the HEP amplitude was significantly different between conditions, the cortical-source differences were tested with the cluster-based permutation *t* test and then the non-parametric permutation test.

## Results

### Subjective and Behavioral Results

#### Gamers vs. Non-gamers

By taking into account the outcomes of the two prior training sessions, gamers started playing at significantly higher levels of the game (*t*(41) = –4.342, *p* < 0.001) and achieved significantly higher levels (*t*(41) = –4.030, *p* < 0.001) than non-gamers. No significant differences were observed in performance between the two groups in terms of total final score (*t*(41) = –1.449, *p* = 0.155) and total final error (*t*(41) = 0.315, *p* = 0.754), which accumulated during playing on the different levels of the game. As shown in Supplementary Table 1 in the Supplementary Data, playing *Thumper* elicited comparably high levels of flow (FSS mean score) in both groups with the value of 4.86 ± 1.03 in non-gamers and 5.34 ± 0.77 in gamers (7 is the maximum value). There were no significant differences between the two groups in self-reported flow (*t*(41) = –1.741, *p* = 0.089) or for the subscales of absorption (*t*(41) = –1.819, *p* = 0.076) and fluency (*t*(41) = –1.324, *p* = 0.193). The estimated duration of the play session, how often participants thought about time, and the passage of time were not significantly different between the two groups. Neither emotional and arousal change nor bodily awareness change before and after playing *Thumper* were significantly different between groups. That is, due to the fact that non-gamers and gamers played on their respective lower and higher performance levels, there were no group differences in related subjective experience of the game.

#### Correlations With Total Flow, Absorption, and Fluency Scores

The correlation coefficients among the total flow/absorption/fluency and other subjective measures considering all participants are shown in Table 1. Since gamers and non-gamers did not differ significantly in subjective variables, the correlation coefficients and related p values for the separate groups are presented in the Supplementary Data in Supplementary Table 2. As shown in Table 1, the more flow participants experienced, the less they thought about time (*r* =–0.481, *p* = 0.001; FDR corrected), and the higher their total game scores was (*r* = 0.466, *p* = 0.002, FDR corrected). The more absorbed participants were in the game, the less they thought about time (ρ = –0.530, *p* < 0.001, FDR corrected), and the faster time passed for them (ρ = 0.423, *p* = 0.005, FDR corrected).

**TABLE 1:** Correlation coefficients and related *p*-Values between the total flow, absorption, and fluency subscales of flow and related variables for all subjects.

Measures & related variables	Total flow (FSS mean score)	Absorption (FSS-Absorption)	Fluency (FSS-Fluency)
	**All *r* (*p*)**	**All *r* (*p*)**	**All *r* (*p*)**
Thinking about time (STSS)	–**0.481^rho^ (0.001^FDR^)**	–**0.530^rho^ (< 0.001)^FDR^**	–**0.394^rho^ (0.009)**
Speed of time passage (STSS)	0.271 (0.079)	**0.423^rho^ (0.005)^FDR^**	0.123 (0.433)
Estimated duration of play session (STSS)	0.101^rho^ (0.519)	0.069^rho^ (0.661)	–0.075^rho^ (0.635)
Total final score in the game	**0.466** (**0.002^FDR^)**	**0.452^rho^ (0.002)^FDR^**	**0.390 (0.010)**
Total final error in the game	–**0.343 (0.024)**	–**0.377^rho^ (0.013)**	–0.232 (0.135)
Bodily awareness (STSS)	–0.160^rho^ (0.307)	–0.167^rho^ (0.285)	–0.149^rho^ (0.342)
Arousal change (SAM-arousal)^1^	–0.032^rho^ (0.839)	0.122^rho^ (0.437)	–0.141^rho^ (0.368)
Valence change (SAM-valence)^1^	0.112^rho^ (0.474)	0.213^rho^ (0.170)	0.019^rho^ (0.904)

*^rho^ Spearman correlation results.*

*^1^Difference between after and before the game session; after-before; significant correlations on the 5% level are marked in bold; FDR = Significant after false-discovery-rate (FDR) adjustment.*

The last two correlations were driven mainly by the gamers. A positive association was also identified between the absorption score and measures of performance. More absorption in the game led to a higher final gaming score (ρ = 0.452, *p* = 0.002, FDR corrected) and lower total errors (ρ = –0.377, *p* = 0.013). Only two correlations appear to be significant on a 1% alpha level for FSS-Fluency. The more fluency participants felt, the less they thought about time (ρ = –0.394, *p* = 0.009; not significant after FDR), and the higher their final game score was (*r* = 0.390, *p* = 0.010; not significant after FDR).

### The Heart-Evoked Potential Measure

**FIGURE 2:**
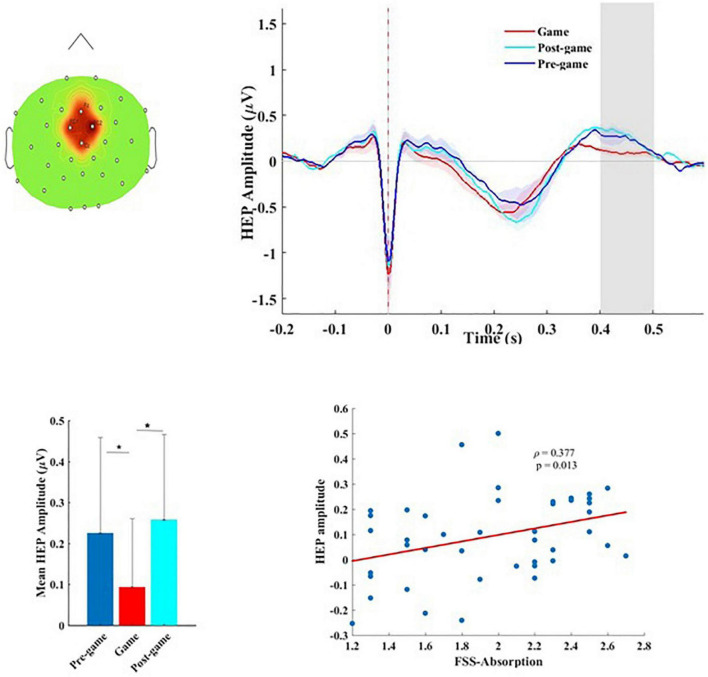
**(A)** Topographical map of the scalp sensors where the distinct heart-evoked potential (HEP) between the game and the pre-/post-game conditions was observed. The red-colored region including FC1, FC2, Fz, and Cz sensors indicates the location of the electrodes contributing to this significant difference, **(B)** the grand average HEP across fronto-central sensors for the three conditions. The shaded area shows the time window in which we observed a significant difference. **(C)** Mean amplitude and the standard deviation of the HEP over the 400–500 ms time window in which the significant difference was observed for the three conditions, **(D)** Flow-absorption score as a function of the HEP amplitude.

Since HEP modulations have been reported in widely distributed scalp electrodes (frontal, central, and parietal sites), as well as a range of latencies between 200 and 600 ms after the R-peak (Pollatos and Schandry, 2004; Babo-Rebelo et al., 2016; Park et al., 2016; Petzschner et al., 2019), we performed a whole brain, whole time-window analysis of the HEPs for the three conditions (pre-game, game, and post-game) using non-parametric permutation tests with 1000 randomizations. Across all subjects, the HEPs significantly differed in the game condition compared to the pre-game and the post-game conditions (considering α < 0.05 with FDR correction) over the fronto-central sensors (Fz, FC1, FC2, Cz) in the time window of 400–500 ms after the R-peak (Figure 2A). The grand average fronto-central HEP waveform is shown for the three experimental conditions in Figure 2B. A *post hoc* analysis revealed that the average HEP amplitude in the time window of 400–500 ms after the R-peak was significantly lower (considering α < 0.05 with FDR correction) over the fronto-central sensors for the game condition compared to the pre-game (*p* < 0.001) and the post-game (*p* < 0.001) conditions (Figures 2B,C). The lower HEP amplitude during the gameplay represents a lower neural response to the heartbeats while playing, which corresponds with our first hypothesis. No significant difference was found for the HEP waveform between the pre-game and the post-game conditions.

The same suppression of the fronto-central HEP amplitude during playing the game was observed within each group. The topographical maps of the average HEP amplitude over the 400 to 500 ms time window after the R-peak for each condition, as well as the corresponding statistical *p* values (derived from non-parametric permutation tests with 1000 randomizations), are separately presented for each group in the Supplementary Figure 1a of the Supplementary Data. As shown in Supplementary Figure 1b, during the game condition the mean HEP amplitude over the 400–500 ms time window in the gamers was slightly higher compared to the non-gamers, which was not statistically significant (*t*(41) = –1.637, *p* = 0.109). To test whether the observed difference in the HEP amplitude was truly time-locked to the heartbeats, the same statistical analysis was conducted repeatedly (100 times), but this time using surrogate R-peaks, which had the same inter-beat interval and variability as the original R-peaks, but were shifted randomly in time. A surrogate analysis performed separately for each group showed no such significant difference as originally found for the real R-peaks, confirming the fact that the differential HEP during the gameplay compared to the resting state is truly locked to the heartbeats.

To test possible associations between the HEP amplitude and flow measures, the mean amplitude of the HEP while playing the game was calculated within the time-window in which a significant difference was detected. No significant association between the HEP amplitude and the level of flow (FSS mean score) was identified across all subjects (*r* = 0.253, *p* = 0.102). The higher the HEP amplitude, the stronger the absorption in the game (ρ = 0.377, *p* = 0.013). This association (Figure 2D) which was not significant within each group (Supplementary Table 4) associated higher absorption in the game with higher cortical processing of cardiac afferents.

#### Neural Sources of the Observed Differential Heart-Evoked Potential for the Game and the Pre-game Conditions

We reconstructed neural sources of the HEP signals for the pregame and the game conditions to identify the cortical regions contributing to the HEP effect observed over the fronto-central scalp electrodes. After identifying the time window for which a significant difference between these two conditions was found in sensor space, a cluster-based permutation test can be applied on the reconstructed cortical currents averaged over that time window. The difference in cortical currents between the pre-game and the game conditions was significant over two large regions, one pronounced over the left supplementary motor area (SMA; BA 6) and left primary motor cortex (BA 4) extending to the left primary somatosensory cortex (BA1, 2,3) and the left posterior cingulate cortex (PCC; cluster size = 1928 vertices, Monte Carlo *p* = 0.002).

**FIGURE 3:**
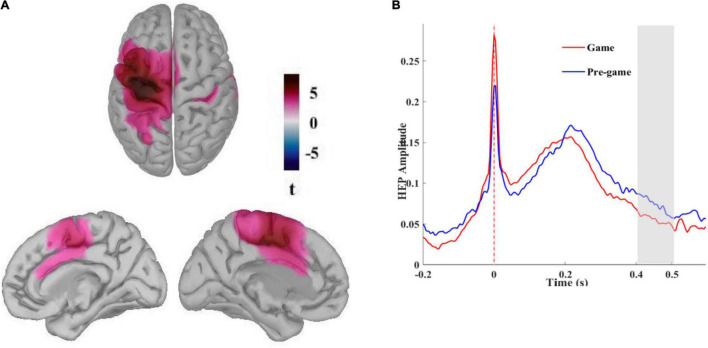
**(A)** Neural sources of the differential HEP for the pre-game and the game conditions. Different activation patterns were identified in the left primary somatosensory and the left supplementary and primary motor cortices extended to the left posterior cingulate cortex (t pertains to permutation test results considering α < 0.01 with FDR correction) stemming from higher activation during the pre-game condition, **(B)** Reconstructed time course of the HEP in the left primary somatosensory cortex grand averaged across all subjects in absolute values of dipole currents for the pre-game and the game conditions. The shaded area shows the time window in which significantly more activation was observed during the pre-game condition.

The other cluster included the right primary somatosensory cortex and the right primary motor cortex (BA4) along with the right PPC (cluster size = 1478 vertices, Monte Carlo *p* = 0.002). We also performed a non-parametric permutation test with FDR correction considering α < 0.01 to identify the most significant regions. As presented in Figure 3A, the HEP neural sources differed significantly between the pre-game and the game condition over the left supplementary and primary motor cortices, the left primary somatosensory cortex extended to the left PCC (Figure 3A). Small regions in the right primary motor cortex, the right primary somatosensory cortex, and the right posterior cingulate were also differently activated between the pre-game and the post-game conditions. The superposition of the statistically different regions with the difference in the absolute values of the cortical currents between the pre-game and the game conditions showed that the activity in the above-mentioned regions during 400 to 500 ms after the R peak was significantly higher in the pre-game condition as compared to the game condition (a video clip demonstrating this source activity difference between the pre-game and the game condition can be found in Supplementary Materials).

**FIGURE 4:**
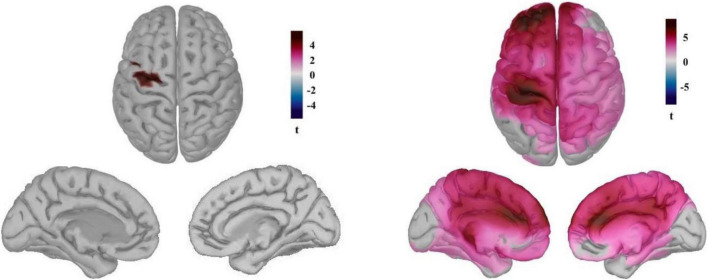
Neural sources of the differential HEP observed for the pre-game and the game conditions for the gamers **(left)** and the non-games **(right)**. (t pertains to permutation test results considering α < 0.05 with the FDR correction).

The time course of the reconstructed neural currents from the primary somatosensory region (Figure 3B) demonstrated lower neural activity locked to the heartbeats in this brain region while playing the game compared to the pre-game condition. We then explored neural sources of the differential HEP observed for the pre-game and the game conditions separately for each group (Figure 4). The permutation test results with an FDR correction considering α < 0.05 showed that in the gamer group only the supplementary motor cortex (the significant region peaked at MNI coordinates –25, –14, 60 with peak *t* = 5.81) was less activated during the game condition compared to the pre-game condition. In non-gamers, though, the left supplementary and primary motor cortex, the left primary somatosensory cortex, the left and right posterior and anterior cingulate cortices, and the left frontopolar prefrontal cortex (BA10; the significant region peaked at MNI coordinates –31, 52, 25 with peak *t* = 8.67) were less activated during the game condition than during the pre-game condition.

Dissimilar neural sources of the observed differential HEP for the gamers and non-gamers may reflect differences in resource allocation to the cortical processing of the heartbeats between these two groups while playing the game vs. the resting state. Similar to the sensor space, the neural sources of the HEP between gamers and non-gamers did not statistically differ in either the pre-game or in the game condition. We also looked at possible correlations between the average neural currents of the identified sources around 400–500 ms after the R-peak in the game condition and subjective flow measures. No associations were identified across the subjects.

#### The Function Between the Heart-Evoked Potential Amplitude and Enhanced Automaticity

Difference in resource allocation to the cortical processing of the cardiac information between two groups while playing the game vs. the resting state raised this question whether the observed relation between the HEP amplitude and absorption was related to the enhanced automaticity experienced during flow state. To address this, we extracted the parietal alpha and explored possible correlations between this measure and the HEP amplitude. According to a previous study (Gevins et al., 1997), higher automaticity leads to higher parietal deactivation (higher parietal alpha). The amplitude of the HEP showed a tendency to associate positively with the parietal alpha power (*r* = 0.314, *p* = 0.04); the higher the HEP amplitude, the higher the parietal alpha power. This finding may suggest that the higher absorption in the game resulted in an increased response to the heartbeats (higher HEP amplitude) through enhanced automaticity.

#### The Function Between the Heart-Evoked Potential Amplitude and Cardiovascular Activity

To explore whether the HEP amplitude is associated with the change in cardiovascular parameters, we searched for possible correlations between the average HEP over the 400–500 ms time window at the fronto-central scalp electrodes and the IBI, the HF- and LF-HRV while playing the game. No associations were identified between the HEP amplitude in this time window and the mentioned cardiac measures during the entire gameplay session. The HEP amplitude was positively associated with the LF- and the HF-HRV power difference between the last and the first 5 min of gameplay (*r* = 0.443, *p* = 0.005; *r* = 0.354, *p* = 0.029, respectively), i.e., the higher the parasympathetic activity recorded during the last 5 min as compared to the first 5 min, the larger the HEP amplitude was.

### Peripheral Measures

All physiological measures were extracted and analyzed for the three experimental conditions of the pre-game (5 min), the game (25 min), and the post-game (5 min). The mean value of the neurophysiological measures for the three experimental conditions and for each group along with the statistical results are presented in the Supplementary Table 3. To examine the pattern of cardiorespiratory activity more precisely during the whole 25-min gameplay, we segmented the recorded physiological signals of the game condition into non-overlapping, 5-min intervals and analyzed the differences between corresponding cardiovascular and respiratory measures for the first and the last intervals. Here we reported the findings regarding all participants. For some differences between gamers and non-gamers we refer to the Supplementary Data (Supplementary Table 4).

#### Respiratory Activity

We analyzed two parameters from the respiration signals: the mean respiration rate (RR-mean), the standard deviation of the respiration rate (RR-STD). The Greenhouse-Geisser correction revealed a significant main effect of condition (pre-game, game, post-game) for the RR-mean (*F*(1.25, 51.28) = 36.70; *p* < 0.001, η^2^ = 0.203) and RR-STD (*F*(1.55, 63.64) = 42.602; *p* < 0.001, η^2^ = 0.314). *Post hoc* tests showed that the mean and variance of the respiration rate were significantly higher during the game compared to the pre-game (*p* < 0.001) and the post-game (*p* < 0.001) conditions. The main group effect (gamers, non-gamers), as well as the interaction between condition and group, was not significant for any of these parameters (see Supplementary Table 4).

There was no association between the RR-mean and flow measures during the whole gameplay session. The respiration-rate difference between the last 5 min and the first 5 min of gameplay was negatively correlated with absorption across all subjects (ρ = –0.424, *p* = 0.005). A slower respiration rate at the end of the gameplay session compared to the beginning (lower sympathetic and higher parasympathetic activity) is related to greater absorption in the game. The variation in the respiration rate (RR-STD) during the whole 25-min game interval was significantly correlated with the total flow (*r* = 0.418, *p* = 0.005), and the absorption sub-scale of flow (ρ = 0.505, *p* < 0.001). These two correlations which were driven mainly by the gamers (Supplementary Table 4) highlighted that the more variation in the respiration rate was associated with more flow and a stronger absorption in the game. The difference in the RR-STD value between the last and the first 5 min of gameplay showed no significant correlations with flow measures.

#### Cardiac Activity

The inter-beat interval (IBI) of the ECG signals differed significantly between conditions (*F*(2, 82) = 8.295; *p* < 0.001, η^2^ = 0.01). *Post hoc* testing revealed a smaller IBI (higher heart rate) during gameplay compared to the pre-game (*p* = 0.005) and the post-game (*p* < 0.001) conditions across all subjects. As shown in Supplementary Figure 2a in the Supplementary Data, the IBI was overall significantly longer for the non-gamers than for the gamers (*F*(1, 41) = 4.68; *p* = 0.03, η^2^ = 0.096) reflecting lower sympathetic activity in non-gamers. The interaction of condition and group was not significant. The data of five participants whose breathing rates were beyond 9–24 cycles per minute (below 9 and above 24 cycles) were excluded from the frequency-domain analysis of the HRV. We found a significant main-condition effect for both the LF-HRV (*F*(2, 72) = 6.21; *p* = 0.003, η^2^ = 0.032) and the HF-HRV (*F*(2, 72) = 16.31; *p* < 0.001, η^2^ = 0.060) measures. *Post hoc* testing showed that both measures were lower during the game compared to the pre-game (*p* = 0.047, *p* < 0.001, respectively) and the post-game (*p* = 0.003, *p* < 0.001, respectively) conditions. No main effect of group or interaction effect was found for these measures.

**FIGURE 5:**
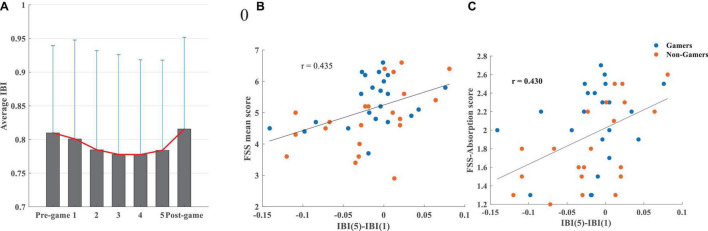
**(A)** The average IBI and its standard deviation during the pre-game, 5-min intervals while playing the game (1, 2, 3, 4, 5), and the post-game conditions, **(B)** association between the difference in the IBI during the last and the first 5 min of gameplay and the FSS mean score, **(C)** association between the difference in the IBI during the last and the first 5 min of the gameplay and the FSS-absorption score.

Figure 5A illustrates that the average IBI continuously declined while playing the game from the first interval up to the third 5-min interval, followed by a plateau for the following 5-min interval, and with a slight increase during the final 5-min interval. During the entire 25 min of gameplay, the shorter the IBI (i.e., the faster heart rate), the higher the total flow score (*r* = –0.325, *p* = 0.033). This effect, which was mainly driven by the non-gamers (Supplementary Table 4), showed that flow was associated with higher sympathetic activity during the whole gameplay period.

Correlations between IBI and absorption/fluency were not significant. The IBI difference between the last and the first 5 min of gameplay was significantly correlated with flow (*r* = 0.435, *p* = 0.003), absorption (ρ = 0.432, *p* = 0.004), and fluency (*r* = 0.354, *p* = 0.020). This relationship, which is mostly driven by non-gamers (Supplementary Table 4), emphasizes that a higher IBI and consequently slower heart rate (lower sympathetic activity) during the last 5 min of playing compared to the first 5 min is positively associated with higher levels of flow (Figure 5B), absorption (Figure 5C), and fluency.

The LF and HF-HRV measures were significantly decreased during gameplay compared to the pre-game and the post-game resting states (see Supplementary Figure 2b). During gameplay, there were no significant associations between LF-HRV and HF-HRV during the game and the total flow experience, absorption, fluency. The HF-HRV power difference between the last and the first 5-min interval of gameplay (Supplementary Figure 2a) showed a positive correlation with the FSS-absorption score (ρ = 0.365, *p* = 0.024). Higher HF-HRV power (higher parasympathetic activity) during the last 5 min compared to the first 5 min of gameplay is associated with higher absorption in the game. No significant correlations were found for the HF-HRV difference and flow or fluency scores. A similar trend for an association was found for the LF-HRV power values showing higher LF-HRV power during the last 5-min interval of gameplay compared to the first, which was associated with higher absorption in the game (ρ = 0.353, *p* = 0.029). These findings show that, although playing the game results in a reduction in parasympathetic activity (lower LF- and HF-HRV), the increase in parasympathetic activity (or, more precisely, less inhibition of parasympathetic activity) at the end of the game leads to higher levels of absorption.

## Discussion

In the present study, we investigated the experience of flow, absorption, and subjective time in gamers and non-gamers while playing the video game *Thumper*. We concentrated on two important aspects of the flow experience, namely the loss of self-referential processing and increased arousal. We used the HEP amplitude as an index of cortical processing of cardiac afferents to evaluate self-referential processing during flow. Associations between the activity of the autonomous nervous system and flow were assessed by evaluating cardiorespiratory measures, including the mean and standard deviation of the respiration rate, the mean IBI, and the average LF- and HF-HRV power.

Our behavioral findings showed that playing *Thumper* elicited comparable levels of flow in both groups with no significant differences in terms of total flow, absorption, or fluency scores. Also, the measures of subjective time did not differ between the groups. We interpret these findings in the way that playing the video game *Thumper* on a level chosen by the participant according to their skill facilitated flow induction. In this way, non-gamers tended to play at relatively lower levels whereas gamers chose relatively higher levels, which made it possible for each group to achieve flow.

The experience of flow was related to the subjective experience of time. The higher the participants’ flow score and the higher the degrees of absorption and fluency they experienced during the game, the less they thought about time. Higher absorption scores were also positively associated with a faster passage of time. These results coincide with those of previous studies that found an association between the experience of flow and subjective time (Keller and Bless, 2008; Rutrecht et al., 2021). Experienced flow, absorption, and fluency were positively correlated with performance, as reflected in the attained final score. This positive association was also reported by several other studies assessing flow (Engeser and Rheinberg, 2008; Jin, 2012; Yun et al., 2017; Rutrecht et al., 2021) and aligns well with the notion of peak performance resulting from the flow experience (Landhäußer and Keller, 2012; Khoshnoud et al., 2020). Task engagement has been related to performance (Matthews et al., 2010). Flow as a positively toned state of high task engagement, signals a cognitive-adaptive process (Matthews et al., 2010) that leads to more effective mobilization of attentional resources to the task (De Sampaio Barros et al., 2018).

### Flow Is Linked to a More Active Brain–Heart Interaction

The HEP amplitude significantly decreased 400–500 ms after the R-peak over the fronto-central scalp electrodes while participants played the game as compared to the pre-game and the post-game conditions. The same HEP difference was observed separately for gamers and non-gamers in the same time range. The use of surrogate R-peaks confirmed that the observed differential HEP during the gameplay session compared to the resting states was actually locked to the heartbeats. This HEP amplitude difference can be considered free from artifactual components because confounding noises, such as cardiac field artifacts, were identified and removed in the preprocessing step using the AMICA. The differential HEP was observed during the time period (400 ms after the R-peak) which is known to be less affected by the cardiac field artifact (Park and Blanke, 2019). The location and latency of the observed differential HEP coincide with previous literature reporting differential HEP between 250 and 500 ms after the R-peak over the fronto-central scalp sensors in modulated self-related processing (Park et al., 2016; Sel et al., 2017; Babo-Rebelo et al., 2019, 2016), and in mental disorders associated with atypical states of self-consciousness, such as depression (Schulz et al., 2015). This lower cortical response to the cardiac afferent while playing may reflect lower self-awareness during gameplay compared to the resting state, in which the sense of self is comparably higher, a typical experience in waiting situations (Jokic et al., 2018). The fact that the fronto-central HEP amplitude was significantly higher during the resting states before and after the game as compared to the game condition reflects higher self-referential processing while waiting. As we have empirically shown, a real waiting period of several minutes is related to stronger negative self-awareness (i.e., boredom) and a slower felt passage of time (Witowska et al., 2020; Alvarez Igarzábal et al., 2021). A waiting period without distraction can be defined as a state with higher levels of self-awareness. The higher HEP amplitude during resting states suggests that bodily signals (such as from the heart) are more readily available during waiting time. This finding corresponds also with a previous study by Wei et al. (2016) that reported a lower fronto-central HEP amplitude during an exteroceptive state (eyes-open resting state) compared to an interoceptive state (eyes-closed resting state). In this regard, playing the game as an intense exteroceptive state led to the lower HEP amplitude compared to the resting states (pre- and post-game), which can be considered relatively more interoceptive.

During the gameplay session, we found a positive correlation between the HEP amplitude and the level of absorption. Individuals who were more absorbed while playing the game exhibited higher HEP amplitudes. Higher levels on the absorption subscale of the flow questionnaire by definition are a strong indicator of flow (Csikszentmihalyi, 1975; Peifer et al., 2014). As formulated by Csikszentmihalyi (1975, p. 39), during flow “your concentration is very complete. Your mind isn’t wandering, you are not thinking of something else; you are totally involved in what you are doing.” Therefore the positive correlation between the absorption subscale of FSS and the HEP amplitude shows a positive association between flow and the HEP. This could be interpreted in the way that flow-absorption during the game necessitated more information from the body, leading to a stronger connection between the brain and the heart. HEP amplitudes around 400–500ms after the ECG R-peak have been mostly investigated and interpreted as correlates of the successive cognitive elaboration of interoceptive information (Baranauskas et al., 2017). In the context of interoceptive predictive coding concepts (Seth et al., 2012; Critchley and Garfinkel, 2018), the brain has been shown to use the interoceptive signals (cardiac information) to successfully predict upcoming exteroceptive events (Park et al., 2014; Pfeiffer and De Lucia, 2017; Banellis and Cruse, 2020). *Thumper* is a dynamic rhythm game that demands high levels of concentration while performing challenging sensorimotor tasks. It is reasonable therefore to assume that precise prediction of sensory stimuli and adequate reactions to them are dependent on information from body states and vice versa, especially since navigating in Thumper necessitates the continuous anticipation of upcoming objects the player has to synchronize with. Therefore, we suggest that the stronger HEP response in more absorbed individuals stems from a more efficient brain-body interaction. This interpretation is substantiated by our findings that more absorbed participants had a higher total final score and a lower total final error in the game (Table 1).

Alpha oscillations has been reported to be important for the efficiency of brain–heart interaction (Luft and Bhattacharya, 2015). Our result showed that higher HEP amplitude is associated with higher parietal alpha power (more deactivation of the parietal cortex). Higher automaticity achieved with increased practice on the task has been linked to higher parietal alpha oscillations (Gevins et al., 1997). We argue that enhanced automaticity during flow may also contribute to the observed function between the HEP amplitude and absorption. The enhanced automaticity during moments of flow through increased reliance on implicit information-processing resources (Dietrich, 2004) may facilitate brain–heart interaction during flow by making more frontal and parietal resources available for the processing of cardiac afferents. It has been reported that enhanced automaticity during flow helps individuals maintain a sustained level of attention so that the increased task demands and challenges can be carried out without a further increase in felt attentional effort (Harris et al., 2017; Yelamanchili, 2018; Khoshnoud et al., 2020).

#### The Heart-Evoked Potential Effect Generated in Regions Associated With Interoceptive Processing

We demonstrated that the HEP effect was generated in regions associated with the processing of internal bodily signals across all participants. The identified neural sources contributing to the observed differential fronto-central HEP between the game and the pre-game conditions covered the left SMA and the left primary motor cortex, extending to the primary somatosensory cortex and the PCC. The activity in these regions was significantly lower during gameplay compared to the pre-game resting state. Since the primary somatosensory cortex and the PCC are two important regions known for the processing of interoceptive signals (Kern et al., 2013; Park and Blanke, 2019), the lower HEP amplitude observed during gameplay corresponds with the lower cortical source currents in regions known for the processing of cardiac signals.

The HEP effect was localized differently in the brain for gamers and non-gamers. While for gamers only the SMA was less activated during the game compared to the pre-game condition, for non-gamers an extended area including the left SMA, the left primary motor cortex, the left primary somatosensory cortex, the left and right PCC and anterior cingulate cortex (ACC), and the left frontopolar prefrontal cortex were less activated during gameplay compared to the pre-game condition. An explanation for this huge difference between gamers and non-gamers in size and location of the identified neural sources contributing to the observed HEP effect may be related to the enhanced skill and automaticity of gamers during the gameplay. For gamers, playing *Thumper* was not such a demanding challenge due to their experience, therefore the HEP source difference before and during the game was relatively smaller. For the non-gamers *Thumper* posed a higher challenge, as they lacked the experience and skills of gamers. Consequently, a larger difference in neural sources was detected between the resting state before the game and the game condition in non-gamers. Dietrich (2004) proposed that higher automaticity during moments of flow can be achieved by increased reliance on implicit information processing (which is supported primarily by the basal ganglia) instead of explicit information processing (which is supported by the frontal and parietal lobes) in the brain. Considering this, the higher automaticity in gamers achieved through the use of more implicit information processing may have allowed them to allocate comparable resources to the cortical processing of the heartbeats during gameplay to the resting state before the game. Non-gamers exhibited less automaticity during the game because of their lack of skills and relied more on the explicit information-processing system (which is based on frontal and parietal resources). This may result in fewer available frontal and parietal resources for the processing of cardiac information while playing the game. Thus, the extended brain regions in frontal and parietal cortex were less activated during gameplay compared to the pre-game condition in these participants in response to the heartbeats.

#### Contribution of Parasympathetic Activity to the Observed Heart-Evoked Potential Effect

Several studies have shown a link between emotional and arousal processes and the HEP amplitude (Fukushima et al., 2011; Shao et al., 2011; Schulz et al., 2013; Luft and Bhattacharya, 2015), but have not always observed a difference in cardiac parameters between experimental conditions in which HEP differences were found (Fukushima et al., 2011; Park and Blanke, 2019). Considering the whole gameplay session, we did not observe any associations between the cardiorespiratory measures and the HEP amplitude. The HEP amplitude was positively correlated with the difference in parasympathetic activity (higher LF and HF-HRV) between the last 5 and the first 5 min of gameplay. This observed positive association shows that the brain–heart interaction is associated with the activation of the vagus nerve at the end of the game compared to the beginning. It has been stated that the vagus nerve is activated during self-transcendent positive emotions (Kitson et al., 2020).

### Flow Is Associated With Increased Sympathetic and Less Inhibition of Parasympathetic Activity

The total flow and absorption levels were positively correlated with variations in the respiration rates (RR-STD), revealing greater variations during the game, higher flow, and stronger absorption in the game. Respiration has been shown to contribute to the short-term modulation of the sympathetic nervous system (Narkiewicz et al., 2006) with a faster respiration rate associated with higher sympathetic activity (Wientjes, 1992; de Manzano et al., 2010). A more varied respiration rate while playing the video game shows greater short-term modulations of the sympathetic nervous system, which may help the respiration system to co-vary with the demands of the game and thereby facilitate the experience of flow and absorption. Total flow was also positively correlated with the average IBI values during the entire game session. The higher the total flow participants reported, the faster their heart rate. These findings align well with the reported positive association between increased sympathetic activity and flow in previous studies (Kivikangas, 2006; de Manzano et al., 2010; Bian et al., 2016). The lower mean-RR (slower respiration rate) and the longer IBI (slower heart rate) during the last 5 min compared to the first were associated with higher absorption in the game. The IBI difference between the last and the first 5-min intervals was also positively correlated with the total flow and fluency levels. These results indicate that higher parasympathetic activity during the last 5 min of gameplay compared to the first 5 min led to the higher flow and absorption levels.

In contrast to studies reporting a relationship between flow and HRV measures (Chanel et al., 2011; Keller et al., 2011; Harmat et al., 2015; Harris et al., 2016; De Sampaio Barros et al., 2018; Kozhevnikov et al., 2018), no significant associations were identified between LF-HRV/HF-HRV and flow measures during the 25 min of gameplay. Parasympathetic activity (including LF and HF-HRV) decreased during gameplay with no significant difference between gamers and non-gamers. This general inhibition of parasympathetic activity reflects increased mental effort while playing as compared to the pre- and post-game resting states. The higher LF-HRV and HF-HRV during the last 5 min of gameplay compared to the first 5 min were positively correlated with the level of absorption in the game among all participants. The reduced inhibition of parasympathetic activity at the end of the game led to higher absorption.

Combining these findings and considering the whole gameplay session, we suggest that flow is associated with increased sympathetic activity manifested by more variable respiration and a faster heart rate. Participants who had less inhibition of parasympathetic activity at the end of the gameplay session compared to the beginning were better able to actually control the heightened sympathetic activity and consequently reported higher flow, absorption, and fluency. According to Porges’ (2001) polyvagal theory, parasympathetic influences are essential for an individual’s successful adaptation to changing environmental demands. Studies have related parasympathetic activity (specifically HRV) to working- memory performance, mental workload, and attention (Veltman and Gaillard, 1998; Hansen et al., 2003). Hansen et al. (2003) reported that a higher resting state parasympathetic activity (higher HF-HRV) is associated with better performance in a working-memory task and continuous performance test. Both increased sympathetic activity and decreased parasympathetic activity have been associated with lower performance (Forte et al., 2019) in a systematic review. Considering the identified positive association between flow and sympathetic activity in this study, relatively less inhibition of parasympathetic activity at the end of the gameplay session compared to the beginning may indicate a higher ability to respond flexibly to the changing demands of the game. Our findings align well with the reported link between the co-activation of sympathetic and parasympathetic activity and the flow experience (de Manzano et al., 2010; Harmat et al., 2015; Tian et al., 2017). The co-activation in our results was demonstrated by increased overall sympathetic activity and decreased inhibition of parasympathetic activity during the last 5 min of gameplay.

All in all, our findings indicate that game-induced flow modulates subjective time perception in terms of less thinking about time and the feeling of a faster passage of time. We showed that the monitoring of cardiac afferent information being processed by the brain can serve as an objective measure to assess the level of absorption in the game. The positive association identified between the fronto-central HEP amplitude and absorption during the game can be interpreted by a better brain–heart interaction that led to a better performance, i.e., a higher final score and a lower total error. Our findings also provide evidence for the relationship between the co-activation of the sympathetic and parasympathetic nervous systems and the flow experience. As an applied perspective of our work, the HEP amplitude could function as a neural marker of flow-absorption in patients with depression and anxiety who have lost their ability to lose track of the self and time. It is known that individuals with Major Depressive Disorder have deficits in emotional self-regulation and also report a drastically slowed down subjective passage of time (Vogel et al., 2018). Further inquiries are needed to assess whether states of flow elicited by games like Thumper ameliorate symptoms of depression by accelerating the subjective flow of time. In one earlier study by Kühn et al. (2018), depressed individuals played the video game Boson X, which has similar characteristics to *Thumper*, for 6 weeks and this reduced rumination and enhanced cognitive abilities. The HEP amplitude thereby could function as neural marker for increased states of flow-absorption during tasks individuals with depression are undertaking. It could reflect improvements of their psychological state.

## Data Availability Statement

The dataset generated for this study can be found on the Open Science Framework website, https://osf.io/djre6/?view_only=1bbd140e80b24c45ac2f8c25123c6d51.

## Ethics Statement

This study was reviewed and approved by the Local Ethics Committee of the Institute for Frontier Areas of Psychology and Mental Health (IGPP_2019_01).

## Author Contributions

SK performed data curation, conducted the analysis, and wrote the first draft. All authors contributed to conceptualization, design of the study, manuscript revision, and approved the submitted version.

## Conflict of Interest

The authors declare that the research was conducted in the absence of any commercial or financial relationships that could be construed as a potential conflict of interest.

## Publisher’s Note

All claims expressed in this article are solely those of the authors and do not necessarily represent those of their affiliated organizations, or those of the publisher, the editors and the reviewers. Any product that may be evaluated in this article, or claim that may be made by its manufacturer, is not guaranteed or endorsed by the publisher.

## Footnotes

1
http://sccn.ucsd.edu/wiki/A08: _DIPFIT
