# Supplementary material for: Brain–Heart Interaction and the Experience of Flow While Playing a Video Game
Source: Front Hum Neurosci. 2022 Apr 28;16:819834. doi: 10.3389/fnhum.2022.819834 (PMC9096496; doi:10.3389/fnhum.2022.819834)
Supplement: Supplementary file 1 [file Data_Sheet_1.PDF]

Brain-Heart Interaction and the Experience of Flow while Playing a Video Game

Supplementary Material

1 Comparisons of Gamers and Non-gamers in subjective ratings of the video game

Supplementary Table 1. The mean values and the standard deviations of different dependent variables for each group and their statistical analysis results

**Measures**<br>**Flow & flow-related variables**	**Mean (S.D.)**		**t or u =**
	**Gamers**	**Non-**	**p =**
**Flow (FSS mean score) [1 … 7]**	5.34 (0.77)	4.86 (1.03)	-1.741
**Fluency (FSS-Fluency) [1 … 6]**	3.26 (0.48)	3.02 (0.68)	-1.324
**Absorption (FSS-Absorption) [1 … 4]**	2.07 (0.43)	1.83 (0.44)	-1.819
**Estimated duration of play session (STSS) [real duration = 25 min. = 1500 sec.]**	1574.6 (484.6)	1715.8(295.5)	283*
**Thinking about time (STSS) [0 … 10]**	1.80 (1.76)	2.88 (2.07)	307*
**Speed of time passage (STSS) [0 … 10]**	7.39 (1.89)	7.00 (1.17)	188.5*
**Bodily awareness (STSS) [1 … 7]**	4.59 (1.53)	4.71 (1.41)	0.274
**Emotional Valence change (SAM) [-4 … 4][1]**	-0.18 (0.73)	-0.47 (0.87)	200*
**Arousal change (SAM) [-4 … 4][1]**	0.86 (0.88)	0.81 (0.92)	223*

* For these measures the results from the Mann-Whitney U test are presented

[1] Difference between after and before the game session; after-before

2 Correlations with the total flow score, the absorption and the fluency scores, separately for the two groups

Supplementary Table 2. Correlation coefficients and related p values between the total flow (FSS mean score) /absorption (FSS-Absorption)/fluency (FSS-Fluency) and other related measures, separately for the two groups.

Measures & related variables	**FSS mean score**<br>r<br>(p)		**FSS-Absorption**<br>r<br>(p)		**FSS-Fluency**<br>r<br>(p)	
	Gamers	Non-Gamers	Gamers	Non-Gamers	Gamers	Non-Gamers
Thinking about time (STSS)	-0.357 [rho]<br>(0.103)	-0.281<br>(0.218)	**-0.432 [rho]**<br>**(0.045)**	-0.384<br>(0.086)	-0.259 [rho]<br>(0.245)	-0.173<br>(0.453)
Speed of time passage (STSS)	0.333<br>(0.130)	0.184<br>(0.424)	**0.459**<br>**(0.032)**	0.282<br>(0.216)	0.121<br>(0.591)	0.094<br>(0.685)
Estimated duration of play session (STSS)	-0.146<br>(0.516)	**0.516 [rho]**<br>(**0.017**)	-0.030<br>(0.894)	**0.454 [rho]**<br>**(0.039)**	-0.205<br>(0.359)	**0.486 [rho]**<br>**(0.026)**
Total final score in the game	0.379<br>(0.082)	**0.464**<br>(**0.034**)	0.412<br>(0.057)	0.418<br>(0.059)	0.236<br>(0.291)	0.427<br>(0.053)
Total final error in the game	-0.356<br>(0.104)	-0.337<br>(0.136)	-0.394<br>(0.070)	-0.421<br>(0.057)	-0.215<br>(0.337)	-0.233<br>(0.310)
Bodily awareness (STSS)	-0.414<br>(0.055)	0.067<br>(0.773)	-0.180<br>(0.422)	-0.181<br>(0.432)	**-0.497**<br>**(0.018)**	0.219<br>(0.341)
Arousal change (SAM-arousal)[1]	-0.172 [rho]<br>(0.443)	0.101 [rho]<br>(0.663)	0.055 [rho]<br>(0.806)	0.208 [rho]<br>(0.366)	-0.290 [rho]<br>(0.190)	0.005 [rho]<br>(0.983)
Valence change (SAM-valence)[1]	0.242 [rho]<br>(0.227)	-0.004 [rho]<br>(0.987)	0.256 [rho]<br>(0.250)	0.109 [rho]<br>(0.637)	0.170 [rho]<br>(0.450)	-0.141 [rho]<br>(0.543)

Significant correlations on the 5% alpha level are marked in bold.
[rho] Spearman correlation results; [1] Difference between after and before the game session; after-before

3 HEP amplitude suppression for Gamers and Non-gamers while playing the game in comparison to the pre- and post-game conditions

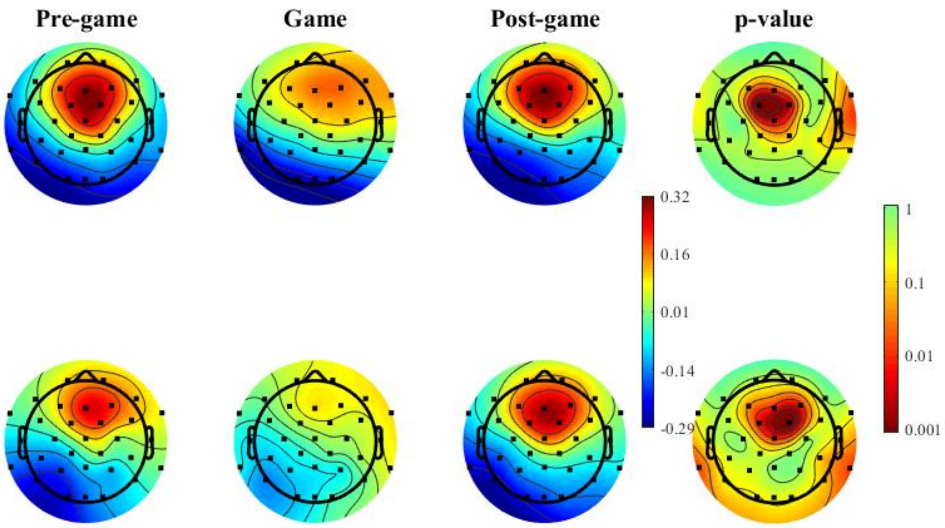

a)

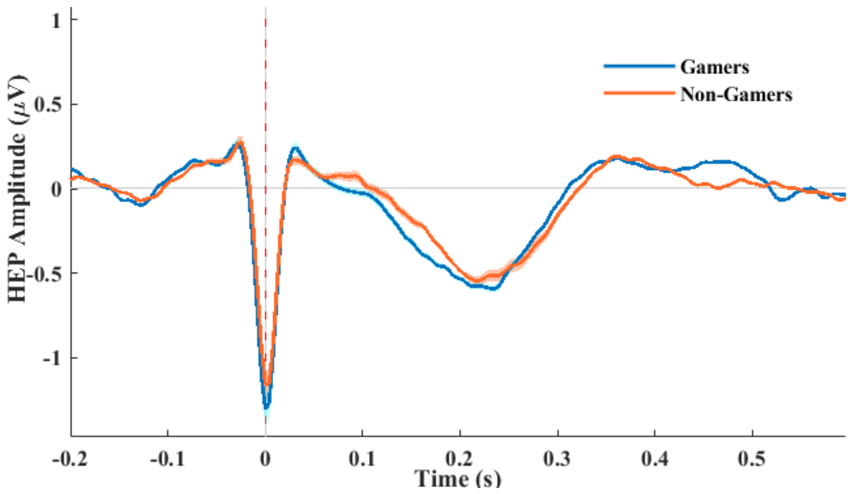

b)

Supplementary Figure 1. Topographical maps of the mean HEP over the 400-500 ms time window after the R-peak for each condition, as well as the corresponding comparison p values for gamers (top) and non-gamers (bottom), b) Mean HEP while playing the game for gamers (blue line) and non-gamers (orange line). There was no significant difference of the HEP amplitude between the two groups.

4 Peripheral measures for the three experimental conditions and both groups

Supplementary Table 3. The mean values of all extracted neurophysiological measures for the three experimental conditions (pre-game, game, post-game) and for each group along with the statistical comparison results

	Experimental conditions						Condition effect	Condition × Group effect	Group effect
	Pre-game		Game		Post-game				
	Gamers	Non-gamers	Gamers	Non-gamers	Gamers	Non-gamers			
**RR-mean (res/min)**	13.0	14.0	17.0	18.5	13.4	14.5	**F = 36.70** ***p* < 0.001*****	F = 0.11 *p* = 0.788	F = 1.41 *p* = 0.24
**RR-STD**	3.3	2.8	5.3	4.9	3.5	3.08	**F = 42.60** ***p* < 0.001*****	F = 0.019 *p* = 0.960	F = 2.21 *p* = 0.145
**IBI (sec)**	0.77	0.84	0.73	0.83	0.77	0.86	**F = 8.311** ***p* < 0.001*****	F = 2.41 *p* = 0.096	**F = 4.68** ***p* = 0.03***
**LF-HRV**	6.9	7.1	6.4	6.94	7.07	7.3	**F = 6.21** ***p* = 0.003*****	F = 0.64 *p* = 0.53	F = 1.62 *p* = 0.209
**HF-HRV**	5.8	6.4	5.07	5.95	5.6	6.2	**F = 16.31** ***p* < 0.001*****	F = 1.13 *p* = 0.327	F = 3.87 *p* = 0.057

Significant correlations are marked in bold**; * *p* < 0.05,** ** *p* < 0.01, *** *p*< 0.001**

5 Correlations between the HEP amplitude/ peripheral measures and flow measures, separately for the two groups

Supplementary Table 4. Correlation coefficients and related p values between the HEP amplitude/ peripheral measures and the total flow (FSS mean score)/absorption (FSS-Absorption)/fluency (FSS-Fluency), separately for the two groups.

Correlated measures	**r (p)**		
	All participants	Gamers	Non-Gamers
The HEP amplitude & FSS-Absorption	**0.377**^{rho} **(0.013)**	0.249 (0.265)	0.347 (0.123)
RR-STD & FSS mean score	**0.418 (0.005)**	**0.455 (0.033)**	0.364 (0.105)
RR-STD & FSS-Absorption	**0.505**^{rho} **(<0.001)**	**0.516 (0.014)**	**0.519 (0.016)**
IBI & FSS mean score	**-0.325 (0.033)**	0.059 (0.793)	-0.395 (0.070)
RR-mean difference between the last and the first 5 minutes & FSS-Absorption	**-0.424**^{rho} **(0.005)**	-0.381 (0.08)	-0.246 (0.228)
IBI difference between the last and the first 5 minutes & FSS mean score	**0.435 (0.003)**	0.388 (0.074)	**0.487 (0.025)**
IBI difference between the last and the first 5 minutes & FSS-Absorption	**0.432**^{rho} **(0.004)**	0.265 (0.233)	**0.597 (0.004)**
IBI difference between the last and the first 5 minutes & FSS-Fluency	**0.354 (0.020)**	0.381 (0.080)	0.345 (0.127)
HF-HRV difference between the last and the first 5 minutes & FSS-Absorption	**0.365**^{rho} **(0.024)**	0.350 (0.154)	0.338 (0.145)
LF-HRV difference between the last and the first 5 minutes & FSS-Absorption	**0.353**^{rho} **(0.029)**	0.349 (0.155)	0.339 (0.144)

Significant correlations on the 5% alpha level are marked in bold.

^{rho} Spearman correlation results

6 Average IBI and HF-HRV/LF-HRV measures separately for Gamers and Non-gamers during three experimental conditions (pre-game, game and the post-game)

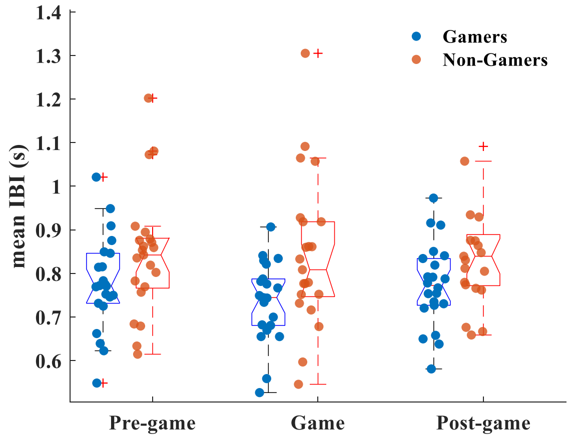

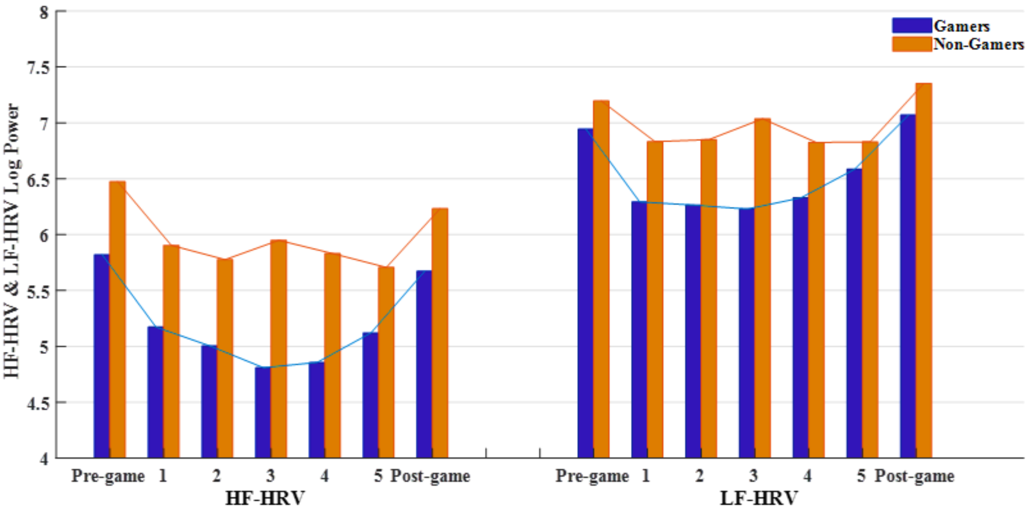

Supplementary Figure 2. a) The average inter-beat interval (IBI) for gamers (blue) and non-gamers (orange) during three experimental conditions (pre-game, game and the post-game), b) Alteration of the HF-HRV (left bar plots) and the LF-HRV (right bar plots) log power during the pre-game, 5-minute intervals while playing the game, and the post-game conditions for the gamers (blue bars) and the non-gamers (orange bars)
